# 5-Amino-3-methyl-Isoxazole-4-carboxylic Acid as a Novel Unnatural Amino Acid in the Solid Phase Synthesis of α/β-Mixed Peptides

**DOI:** 10.3390/molecules27175612

**Published:** 2022-08-31

**Authors:** Urszula Bąchor, Agnieszka Lizak, Remigiusz Bąchor, Marcin Mączyński

**Affiliations:** 1 Department of Organic Chemistry and Drug Technology, Faculty of Pharmacy, Wroclaw Medical University, 50-556 Wroclaw, Poland; 2 Faculty of Chemistry, University of Wroclaw, 50-383 Wroclaw, Poland

**Keywords:** non-proteinogenic amino acid, α/β-mixed peptides, β-amino acids, peptide synthesis, isoxazole, ESI-MS

## Abstract

The hybrid peptides consisting of α and β-amino acids show great promise as peptidomimetics that can be used as therapeutic agents. Therefore, the development of new unnatural amino acids and the methods of their incorporation into the peptide chain is an important task. Here, we described our investigation of the possibility of 5-amino-3-methyl-isoxazole-4-carboxylic acid (AMIA) application in the solid phase peptide synthesis. This new unnatural β-amino acid, presenting various biological activities, was successfully coupled to a resin-bound peptide using different reaction conditions, including classical and ultrasonic agitated solid-phase synthesis. All the synthesized compounds were characterized by tandem mass spectrometry. The obtained results present the possibility of the application of this β-amino acid in the synthesis of a new class of bioactive peptides.

## 1. Introduction

Isoxazoles are heterocyclic compounds with a broad spectrum of targets and high biological activity. They are useful in the development of new therapeutic agents with an increased potency and lower toxicity [1,2]. Their properties have been tested due to their anticancer, anti-inflammatory, and antibacterial activities [3,4,5]. The growing popularity of compounds with isoxazole moiety may be illustrated by the great list of isoxazole-containing drugs approved by the FDA and EMA, such as parecoxib [6], micafungin [7], and leflunomide [8]. In addition to their use as therapeutics, isoxazoles are also used in the organic synthesis of novel classes of compounds, including peptides. 5-amino-3-methyl-isoxazole-4-carboxylic acid is an example of a bifunctional isoxazole derivative that includes in its structure both an amino and carboxylic group. It is a type of β-amino acid in which the amino group is present on the carbon atom at the β position to the carboxy group; thanks to this it can be considered a non-proteinogenic amino acid. Such unnatural amino acids are becoming an increasingly important tool in drug discovery and has led to an increase in the number of synthetic methods for the preparation of that class of compounds [9,10,11]. Peptidomimetics are still of a great interest in the development of new biologically active molecules [12]. The therapeutic application of peptides obtained from natural amino acids is limited due to the problems connected with the low stability against proteolysis. One of the strategies to overcome this issue is the use of a compound that can mimic the action of a particular peptide [13]. The hybrid α/β-mixed peptides show great promise as peptidomimetics that can be used as therapeutic agents [14]. They can be obtained in a simple way using a popular technique of peptide synthesis described already in the 1960s by Merrifield [15].

Non-proteinogenic amino acids can be obtained by modifying proteinogenic amino acids or related compounds [16], but many are also formed as secondary metabolites in bacteria, fungi, or marine organisms, such as MeBmt ((4*R*)-4-[(*E*)-2-butenyl]-4*N*-dimethyl-l-threonine) [17] present in antifungal products and in cyclosporin A, which is a strong immunosuppressive agent commonly used in post-transplant therapy. Unnatural amino acids (UAAs) are often used as building blocks in drug discovery and as reagents for important drug-like compounds (i.e., benzodiazepines) [18]. One of the best examples is 2*R*, 3*S*-*N*-benzoyl-3-phenylisoserine, which is a component of paclitaxel, an anticancer drug used to treat breast, ovarian, and lung cancer, typically in combination with cisplatin or an anthracycline. It shows an antagonistic effect on microtubules and causes mitosis arrest, which leads to cell death [19,20]. Peptides are increasingly entering the world of pharmacologically active substances in medicine, mainly due to their non-complex preparation method, often based on solid-phase synthesis.

There are many approved drugs and biologically active molecules that are made up entirely of a non-proteinogenic amino acid. A good example is 3,4-dihydroxy-l-phenylalanine, or L-DOPA, also called levodopa, used in Parkinson’s disease [21,22,23]. Naturally occurring peptides display desirable medicinal properties but are often limited in application due to their rapid proteolysis. Various modifications were developed to address these challenges. Different approaches are used to improve the metabolic stability or therapeutic potential; one of them is the incorporation of unnatural amino acids analogs or peptidomimetics or to apply a structural modification. Nowadays, peptides containing non-proteinogenic amino acids are a highly explored group of compounds due to their biological properties, including their potent antifungal activity [24]. Blaszczyk and co-workers [25] presented the potent Human Arginase 1 (a target in cancer immunotherapy) inhibitors based on the 2(*S*)-amino-6-boronohexanoic acid, another unnatural amino acid, as alternatives for previously described compounds, which were based on the aliphatic and aromatic rings-containing non-proteinogenic amino acids [26]. Potent and long-acting single-chain peptide mimetics of human relaxin-2 for cardiovascular diseases based on the containing amino isobutyric acid were also presented [27].

Due to the presented biological activities and physicochemical properties of unnatural amino acids, the development of new methods of their incorporation into the peptide chain via solid phase peptide synthesis is an important task. Recently, we described the method of mimosine-containing peptide synthesis without any side protection [28]. The proposed strategy allowed us to insert this unnatural amino acid residue at any endo-position within a peptide sequence. Additionally, we synthesized and analyzed the chemical properties of a series of peptomers [29], peptide hybrids [30], and isoxazole-linked derivatives [31,32].

In this work, we analyzed the possibility of a 5-amino-3-methyl-isoxazole-4-carboxylic acid (AMIA) application in classical and ultrasonic agitated solid-phase peptide synthesis as a new β-amino acid. The proposed method may open possibilities of new unnatural amino acids’ incorporation, presenting various biological activities, into the peptide chain.

## 2. Results and Discussion

**Figure 1:**
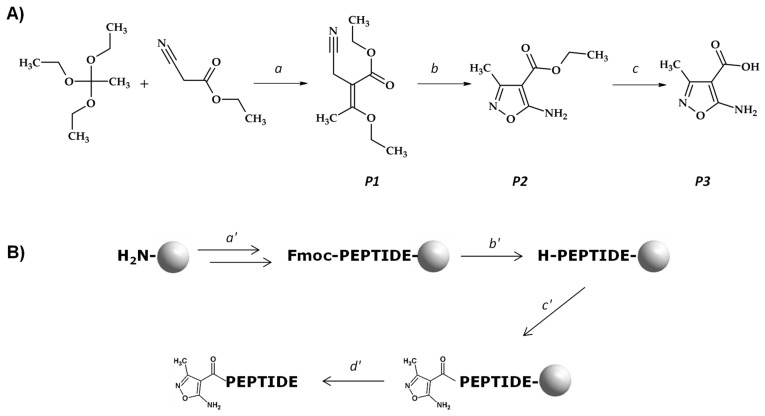
(**A**) Schematic presentation of the AMIA synthesis. Conditions: (a) DMAP, 110 °C, ethanol removement (*P1*); (b) EtONa, 0 °C; NH_2_OH∙HCl in EtOH (*P2*); (c) NaOH (aq); HCl to pH 3 (*P3*). (**B**) Application of AMIA in the solid-phase peptide synthesis. Conditions: (a′) SPPS-Fmoc-Aaa (3 eq), HATU (3 eq), DIPEA (6 eq), 2 h, RT; (b′) 25% piperidine in DMF (2 × 10 min), RT; (c′) AMIA coupling (3 eq), HATU (3 eq), DIPEA (6 eq), ultrasonic agitation 3 × 15 min; (d′) TFA/H_2_O/TIS (95:2.5:2.5; *v*/*v*/*v*, 2 h, RT). Grey ball indicates Rink Amide MBHA resin.

The main goal of this work was to investigate the possibility of a 5-amino-3-methyl-isoxazole-4-carboxylic acid (AMIA) application in the efficient synthesis of a peptide on solid support, according to the presented scheme (Figure 1).

In the case of standard Fmoc-peptide synthesis, commercially available amino acid residues contain a blocked main chain amino group and side chain functionalities to limit the possibility of side products’ formation. Therefore, to protect the amino group in the analyzed compound, we decided to use a similar procedure of the Fmoc group introduction as proposed previously by us for mimosine as another unnatural amino acid [28]. In this reaction, the precipitate of the Fmoc-protected amino acid should be obtained; however, its formation was not observed. The ESI-MS analysis of the supernatant revealed the presence of the signal at *m*/*z* 365.116, corresponding to the protonated Fmoc-AMIA-OH derivative with the intensity at the level of noise. Additionally, the HPLC analysis of the solution revealed a very low intense signal, corresponding to the final product. It was reported that the amino group presented in the 5-amino-3-methyl-isoxazole-4-carbohydrazide ring acts as the imine group [33]. Detailed NBO (natural bond orbital) analysis has provided detailed insight into donor-acceptor interactions and the nature of bonding in 5-amino-3-methyl-isoxazole-4-carbohydrazide. Also, the conformational search for possible conformers and tautomers of AMIA has been presented. The X-ray data were compared with the calculated ones with the B3LYP/6311++G (*df,pd*) theory level [34]. It should be mentioned that the total percentage of the AMIA resonance structure with a double C=N bond is 34.19%; additionally, a strong intramolecular hydrogen bond between one of the hydrogens of the amino group and oxygen from the carbonyl group was observed, which stabilizes the structure, although it was shown that the amino group of AMIA and its derivatives remains nonreactive in reactions with acyl- and alkyl donors. This phenomenon may allow the introduction of the applied non-proteinogenic amino acid (AMIA) only at the *N*-terminus of the synthesized peptide, which is not considered a significant limitation. However, in further analysis, we decided to check whether this phenomenon exists by trying to modify the amino group of the peptidyl resin containing the *N*-terminal AMIA residue. Additionally, the imidic character of the amino group may affect the ninhydrin test (Kaiser test), which, in the presence of the secondary amino group, gives a yellow-dark colour to the resin beads. However, this reaction can still be used for the analysis of the AMIA coupling efficiency to the amino group located on the peptidyl resin. Therefore, in our study, we decided to omit the amino group protection and apply non-protected AMIA in the peptide synthesis. To analyze the effectiveness of AMIA coupling in the solid-phase synthesis, a coupling reagent in the form of HATU (O-(7-azabenzotriazol-1-yl)-*N*,*N*,*N*′,*N*′-tetramethyluronium hexafluorophosphonate), and two different solid phase synthesis approaches were applied. First was the classical procedure, which required a 2 h coupling reaction with the coupling step repetition. The second was the solid-phase synthesis, accelerated by ultrasonic agitation, which allowed a tenfold reduction of the time of synthesis in the Fmoc strategy and improved the purity of the final product [35]. The common ultrasonic bath available in most laboratories all over the world was used.

To analyze the possibility of non-protected AMIA coupling to the amino group of the peptide located on the resin (Table 1), the following model compounds were synthesized and characterized by ESI-MS and HPLC: H-DVYT-NH_2_ ([M + H]^+^ = 496.240 Da), H-EAAA-NH_2_ ([M + H]^+^ = 360.187 Da), and H-PPPP-NH_2_ ([M + H]^+^ = 503.297 Da). The DVYT sequence, described previously as an immunosuppressive fragment of the HLA-DQ molecule [36], was selected as a model due to the presence of various side chain functionalities. In this work, the abbreviations and designations, in accordance with the recommendations of the European Peptide Society, were used as follows: D-aspartic acid; V-valine; Y-tyrosine; T-threonine; E-glutamic acid; A-alanine; P-proline [37]. Such models allow a wider analysis of the possibility of AMIA’s application in the solid-phase peptide synthesis. The synthesized peptides were cleaved from the resin using a TFA/water/TIS (95/2.5/2.5 *v*/*v*/*v*) mixture and analyzed both by the ESI-MS and HPLC techniques.

### 2.1. AMIA Coupling to the Model Peptidyl Resin with the H-DVYT Sequence (1)

**Figure 2:**
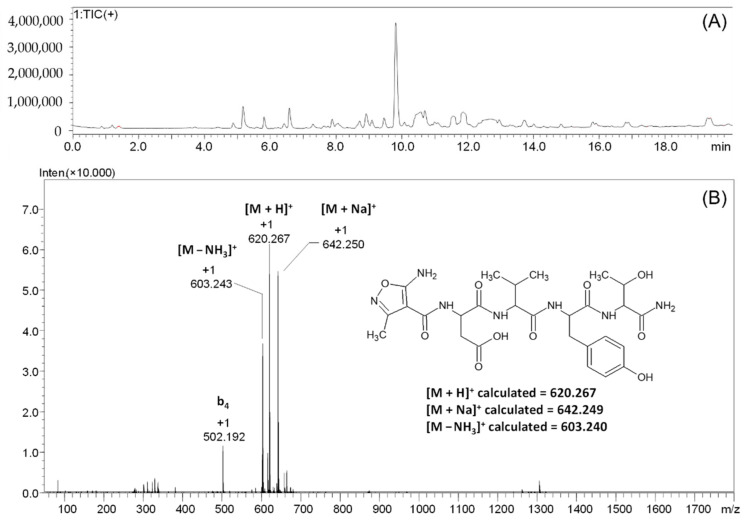
LC-MS analysis of crude product **1**. Total ion current (**A**) and mass spectrum (**B**) of the signal characterized by the retention time of 9.82 min.

In the first experiment, the peptidyl resin H-DVYT-RINK was reacted with the AMIA using both the classical procedure (2 h coupling) and ultrasonic agitation for 15 min. In both cases, the ninhydrin test revealed the presence of slightly purple resin beads; therefore, the coupling procedure was repeated. This allowed the effective coupling of the used non-proteinogenic amino acid to the peptide located on the resin. After the reaction product was cleaved from the solid support, the sample was lyophilized, and the dry residue was analyzed by LC-MS. The obtained results are presented in Figure 2.

In the obtained chromatogram (Figure 2A), the signal, characterized by a retention time of 9.82 min, is present. For the signal characterized by the retention time of 9.82 min, the mass spectrum presented on panel B was recorded (Figure 2B). On the obtained spectrum, the signal characterizing of the [M + H]^+^ ion of the protonated form of the final peptide at *m*/*z* 620.267 was identified. The signal at *m*/*z* 642.250 characterizes the sodium adduct of the peptide [M + Na]^+^. The loss of the ammonia molecule from the tested peptide can also be noticed, as indicated by the signal at *m*/*z* 603.243, characterizing the [M-NH_3_]^+^ ion. The signal at *m*/*z* 502.192 characterizes the b_4_ fragment ion, resulting from the fragmentation in the ion source.

**Figure 3:**
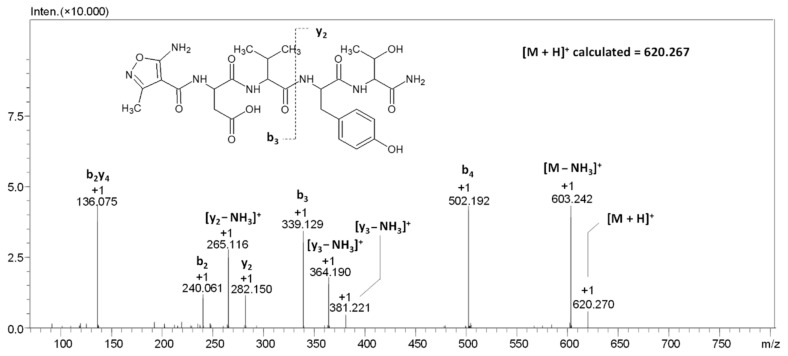
ESI-MS/MS spectrum of compound **1**. Parent ion *m*/*z* 620.270, collision energy 20–40 eV.

To confirm the chemical structure of the compound characterized by the signal at *m*/*z* 620.270, the MS/MS analysis at different collision energies (20–40 eV) was per-formed. The obtained ESI-MS/MS spectrum is presented in the Figure 3.

The obtained chromatograms and mass spectra clearly indicate a lack of additional signals corresponding to the non-modified compound (Figure 3). Therefore, in the analysis of the possibility of AMIA coupling to other model peptides, we decided to apply solid phase synthesis accelerated by ultrasonic agitation for 15 min and repeated three times.

### 2.2. AMIA Coupling to the Model Peptidyl Resin with the H-EAAA Sequence (2)

**Figure 4:**
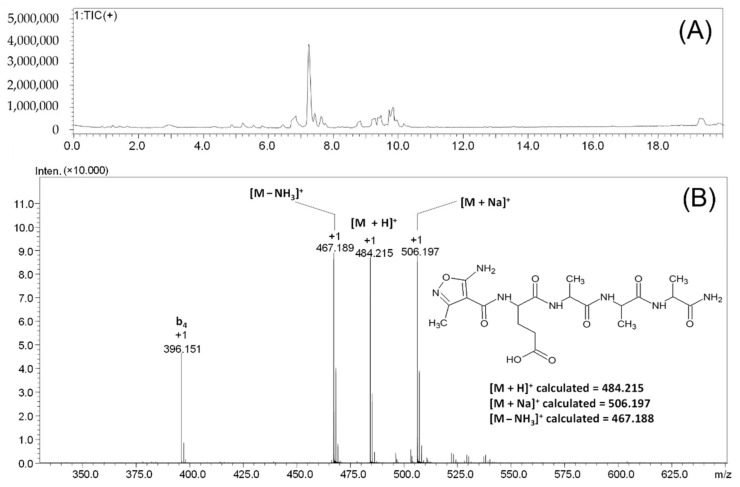
LC-MS analysis of crude product **2**. Total ion current (**A**) and mass spectrum (**B**) of the signal characterized by the retention time of 7.25 min.

The coupling of the AMIA amino acid to the *N*-terminal glutamic acid residue and the fragmentation spectra of the obtained peptide were analyzed. The applied three steps coupling of the AMIA derivative to the model peptidyl EAAA-RINK resin resulted in the formation of a fully modified peptide (Figure 4).

In the obtained chromatogram (Figure 4A), one signal, characterized by a retention time of 7.25 min, was observed. For this signal, the mass spectrum presented in Figure 4B was recorded. The signal characterizing of the [M + H]^+^ ion of the protonated form of the peptide at *m*/*z* 484.215 can be observed. The signal at the value of *m*/*z* 506.197 characterizes the sodium adduct of the obtained peptide [M + Na]^+^. The loss of the ammonia molecule can also be seen, as indicated by the signal at *m*/*z* 467.189, characterizing the [M − NH_3_]^+^ ion. The signal at *m*/*z* 396.151 characterizes the fragmented b_4_ ion that was formed due to the ion source conditions.

**Figure 5:**
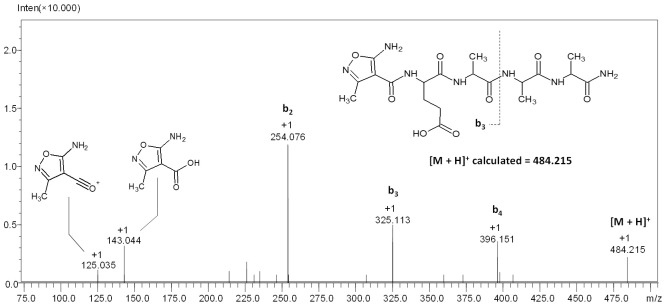
ESI-MS/MS spectrum of compound **2**. Parent ion *m*/*z* 484.215, collision energy 20–40 eV.

To confirm the chemical structure of the compound characterized by the signal at *m*/*z* 484.215, ESI-MS/MS analysis was performed, and the obtained results are presented in Figure 5.

On the obtained spectrum (Figure 5), five signals from the fragment ions can be observed. The signal at *m*/*z* 396.151 characterizes the b_4_ ion; the signal at *m*/*z* 325.123 characterizes the b_3_ ion; the signal at *m*/*z* 254.076 characterizes the b_2_ ion with the highest intensity; the signal at *m*/*z* 143.044 characterizes the ion of the protonated form of the AMIA moiety; the signal at *m*/*z* 125.035 characterizes the AMIA oxonium ion. The characteristic higher intensity of the signal corresponding to the b_2_ ion results from the glutamic acid effect. Peptides containing aspartic or glutamic acid may form b ions, which may undergo cyclization on the C-terminus. As a result of the proton transfer from the carboxyl group to the nitrogen atom of the amide bond, a five-membered ring of succinimide anhydride is formed [38].

### 2.3. AMIA Coupling to the Model Peptidyl Resin with the H-PPPP Sequence (3)

**Figure 6:**
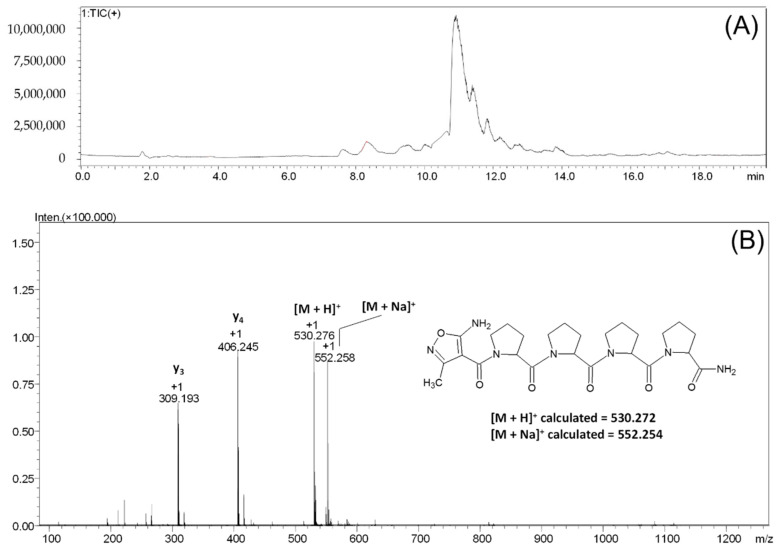
LC-MS analysis of crude product **3**. Total ion current (**A**) and mass spectrum (**B**) of the signal characterized by the retention time of 10.90 min.

In another experiment, we checked the possibility of AMIA coupling to the amino group of the tetraproline peptide attached to the Rink resin. The AMIA amino acid coupling was accelerated by ultrasonic agitation for 15 min. The procedure was repeated three times. The obtained results of the LC-MS analysis of the product cleaved from the resin are presented in Figure 6.

On the obtained chromatogram (Figure 6), two signals, characterized by the retention time of 10.38 and 10.90 min, respectively, can be observed. For the signal characterized by the retention time of 10.90 min, the mass spectrum presented on panel B was recorded (Figure 6B). The signal characterizing the [M + H]^+^ ion of the protonated form of the final peptide at the *m*/*z* 530.276 can be observed on the obtained spectrum. The signal at *m*/*z* 552.258 characterizes the sodium adduct of the tested peptide [M + Na]^+^. The signal at *m*/*z* 406.245 characterizes the y_4_ fragmentation ion; the signal at *m*/*z* 309.193 characterizes the y_3_ fragmentation ion formed in the ESI source during ionization.

For the signals characterized by the retention time of 10.38 min, the recorded ESI-MS spectrum revealed the presence of the non-modified tetraproline peptide used as a model, which the chemical structure of was confirmed using an MS/MS experiment for the parent ion at *m*/*z* 406.249.

**Figure 7:**
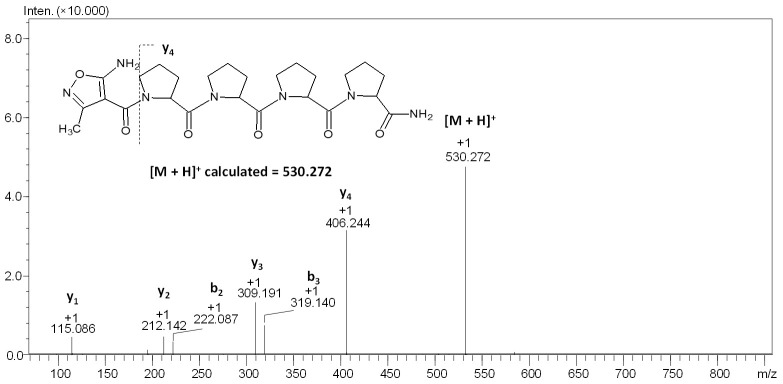
ESI-MS/MS spectrum of compound **3**. Parent ion *m*/*z* 530.272, collision energy 20–40 eV.

On the obtained MS/MS spectrum of peptide **3** (Figure 7), the signal of the parent ion at *m*/*z* 530.272 and nine signals from fragment ions with the *m*/*z* of 406.244, 319.140, 309.191, 222.087, 212.142, 115.086, and 98.060 correspond to the following structures: y_4_, b_3_, y_3_, b_2_, y_2_, y_1_, and y_1_-NH_3_ were identified. The obtained results clearly confirmed the presence of the proline effect in the collision-induced dissociation experiment.

Under CID conditions, the fragmentation of protonated peptides containing a proline residue in the sequence leads to the presence of intense signals corresponding to the formation of the y-type fragment ions, which were produced by dissociating the amide bond at the proline residue [39]. The proline effect is explained by the high affinity of protons to the tertiary amide of the proline residue [39,40]. Moreover, computational studies have shown that during the fragmentation of protonated peptides, unstable b fragments may also form. In this case, proline is at the C-terminus of the peptide chain, and formed b ions have a bicyclic structure [41]. The presence of the proline effect, resulting from the high proton affinity to the basic tertiary amide of the proline residue, explain, also, the lower electron density, and thus basicity, of the amino group of the AMIA moiety.

### 2.4. Coupling of Fmoc-Ala-OH to the Model Peptidyl Resin with the H-AMIA-PPPP Sequence (4)

**Figure 8:**
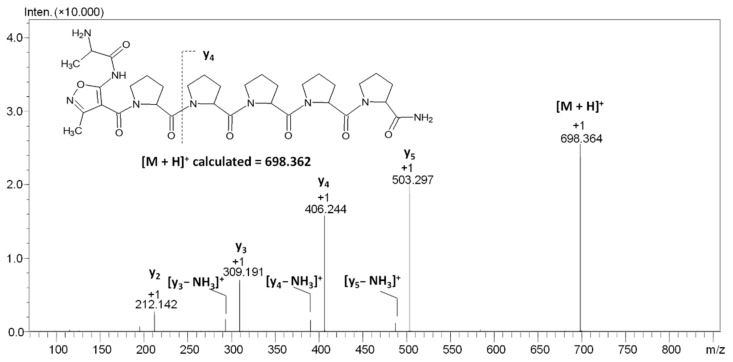
ESI-MS/MS spectrum of compound **4**. Parent ion *m*/*z* 698.364, collision energy 20–40 eV.

Lower nucleophilicity of the AMIA amino group, resulting from its imidic character and electron density delocalization through the isoxazole ring, may affect its reactivity with acyl donors, i.e., in the coupling reaction with other amino acids. To test this hypothesis, we decided to perform the experiment in which the peptidyl resin containing the AMIA moiety at the *N*-terminus in the form of AMIA-PPPPP-RINK was coupled with the Fmoc-Ala-OH, using both classical solid-phase synthesis and accelerated by ultrasonic agitation. The detailed analysis revealed that there is a possibility of AMIA amino group acylation; however, it requires six times the coupling, accelerated by ultrasonic agitation (Figure 8). The classical synthesis did not provide the preparation of the final product.

To confirm the chemical structure of the compound characterized by the signal at *m*/*z* 698.364, ESI-MS/MS analysis at different values of the collision energy (20–40 eV) was performed (Figure 8).

**Figure 9:**
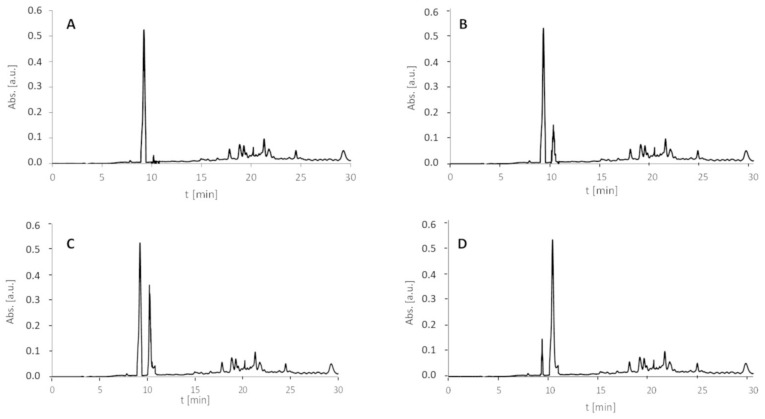
HPLC chromatograms (detection at 210 nm) of crude peptide 1 (**A**) after classical AMIA coupling to the DVYT-peptidyl resin and after ultrasonic-agitated coupling (**B**) within 15 min; (**C**) 2 × 15 min and (**D**) 3 × 15 min.

On the obtained spectrum (Figure 8), the signal of the parent ion [M + H]^+^ and eight signals from the fragment ions can be observed. The signal at *m*/*z* 503.297 characterizes the y_5_ ion, the signal at *m*/*z* 486.271 characterizes the y_5_-NH_3_ ion, the signal at *m*/*z* 406.244 characterizes the y_4_ ion, the signal at *m*/*z* 389.218 characterizes the y_4_-NH_3_ ion, the signal at *m*/*z* 309.191 characterizes the y_3_ ion, the signal at *m*/*z* 292.165 characterizes the y_3_-NH_3_ ion, and the signal at *m*/*z* 212.142 characterizes the y_2_ ion. Once again, the characteristic proline effect was observed. The signal corresponding to the [M + H]^+^ ion of the H-A-AMIA-PPPPP-NH_2_ observed on the ESI-MS spectrum was very low. The MS/MS analysis required the modification of the apparatus parameters to confirm the chemical structure of the obtained product. Although such modification was observed, it can be concluded that the coupling reaction of Fmoc-Ala-OH to the AMIA-PPPPP-RINK peptidyl resin is not efficient and is expensive, time-consuming, and may lead to the side reaction. The performed experiment, despite the fact that it has shown the possibility of such a reaction, is irrelevant from the synthetic point of view. To present the effect of ultrasonic agitation on the AMIA coupling to the DVYT peptidyl resin, we compared the HPLC chromatograms (with detection at 210 nm) of peptide 1 obtained after classical (Figure 9A) and ultrasonic-agitated AMIA coupling after 15 min (Figure 9B), 2 × 15 min (Figure 9C), and 3 × 15 min (Figure 9D).

**Figure 10:**
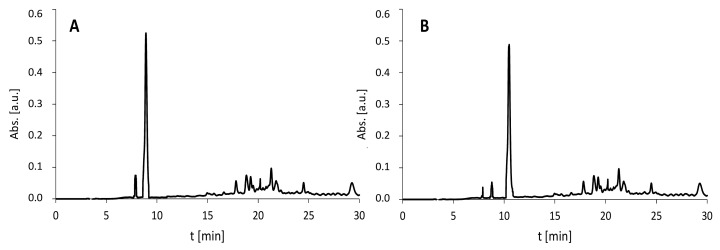
HPLC chromatograms (detection at 210 nm) of (**A**) peptide 2 and (**B**) peptide 3 after ultrasonic-agitated coupling within 3 × 15 min. (**A**) The model peptide with H-EAAA-NH_2_ and peptide 2 (H-AMIA-EAAA-NH_2_) were characterized by the retention time at 7.9 min and 8.9 min, as follows. (**B**) The model peptide with H-PPPP-NH_2_ and peptide 3 (H-AMIA-PPPP-NH_2_) were identified in the fractions characterized by the retention time at 8.8 min and 10.5 min, as follows.

The model peptide with an H-DVYT-NH_2_ sequence was characterized by the retention time at 9.2 min, whereas the H-AMIA-DVYT-NH_2_ derivative was at 10.1 min. Additionally, the HPLC chromatograms (with detection at 210 nm) of peptides 2 and 3 (Figure 10) obtained after 3 × 15 min AMIA coupling by ultrasonic agitation present the advantage of the proposed method.

Microwave-assisted peptide synthesis is one of the most common tools, as it may significantly reduce the reaction time and improve its efficiency [42]. Therefore, we decided to apply it in the AMIA coupling to the peptidyl-resin; however, the decreased stability of the 5-amino-3-methyl-isoxazole-4-carboxylic acid was observed during the microwave irradiation and heat generation. Therefore, as an alternative, we decided to use ultrasonic agitation.

## 3. Materials and Methods

### 3.1. Reagents

All solvents and reagents were used as supplied. Fmoc amino acid derivatives were purchased from Novabiochem (Billerica, MA, USA). O-(7-azabenzotriazol-1-yl)-*N*,*N*,*N*′,*N*′-tetramethyluronium hexafluorophosphonate (HATU), the Rink Amide MBHA resin (0.69 mmol/g), Fmoc-Gly-Wang resin (0.73 mmol/g), and trifluoroacetic acid (TFA) were obtained from IrisBiotech (Marktredwitz, Germany). Solvents for peptide synthesis (*N*,*N*-dimethylformamide (DMF), dichloromethane (DCM), and (*N*-ethyldiisopropylamine (DIEA)) were obtained from Sigma Aldrich (St. Louis, MO, USA).

### 3.2. 5-Amino-3-Methyl-Isoxazole-4-Carboxylic Acid Synthesis

The 5-amino-3-methyl-4-isoxazolecarboxylic acid was prepared via three-steps synthesis (Figure 1A). Ethyl 2-cyano-3-ethoxybut-2-enoate’s preparation (*P1*): Triethyl orthoacetate was mixed with ethyl cyanoacetate (1/1 mol/mol) in a round bottom flask, and the catalytic amount of DMAP was added. The mixture was heated to 110 °C with the simultaneous removal of ethanol formed during the reaction. Then, the mixture was cooled down, and the obtained precipitate was filtered and washed with a 10% HCl solution. Ethyl 5-amino-3-methyl-isoxazole-4-carboxylate’s (*P2*) preparation: Intermediate *P1* was dissolved in ethanol and added to the mixture of EtONa and NH_2_OH∙HCl in EtOH. The mixture was mixed for 24 h at room temperature. Then, the excess ethanol was evaporated. The obtained precipitate was filtered, washed with water, and dried. 5-amino-3-methyl-4-isoxazolecarboxylic acid’s preparation (*P3*): The solid intermediate *P2* was dissolved in a 10% NaOH solution and heated to 70 °C. Then, the mixture was cooled down, and the HCl was added to pH 4. The obtained precipitate was filtered, washed with water, and dried.

### 3.3. Fmoc-AMIA synthesis

The synthesis of the Fmoc derivative of the aminoisoxazole acid was carried out by the method described by Upadhyay et al. [43]. Briefly, AMIA (200 mg, 1.4 mmol) and sodium carbonate (Na_2_CO_3_) (220 mg, 2.1 mmol) were dissolved in distilled water (3 mL). Fmoc-Osu (500 mg, 1.48 mmol), dissolved in 3,6 mL of 1,4-dioxane, was added dropwise to the solution and stirred for 20 h at room temperature. Next, 12 mL of Na_2_CO_3_ (0.1 M) was added. The mixture was stirred for 7 h at 26 °C and was then filtered and washed with 20 mL of ethyl acetate to remove the excess Fmoc-Osu and by-products. The water fraction was kept in an ice bath and adjusted to pH 4.0 using 6 N HCl and incubated overnight at 4 °C. The resulting precipitate was filtered, washed with distilled water, and dried under reduced pressure to give Fmoc-AMIA. The obtained product was analyzed by ESI-MS.

### 3.4. Peptide Synthesis

The synthesis of model peptides on the MBHA-Rink amide resin was performed manually in polypropylene syringe reactors (Intavis AG) equipped with polyethylene filters, according to a standard Fmoc (9-fluorenylmethoxycarbonyl) solid-phase synthesis procedure [44] using 30 mg of Rink Amide MBHA Resin (loading 0.69 mmol/g) for the synthesis of each peptide. The coupling was performed using an Fmoc-protected amino acid (3 eq), AMIA (3 eq), HATU O-(7-azabenzotriazol-1-yl)-*N*,*N*,*N*′,*N*′-tetramethyluronium hexafluorophosphonate (3 eq), and DIPEA (*N,N*-diisopropylethylamine) (6 eq) for 2 h at room temperature. Ultrasonic agitated coupling of AMIA was performed using 3 eq of AMIA, HATU (3 eq), and DIPEA (6 eq) for 15 min. The reaction was repeated three times. The efficiency of the peptide bond formation was controlled by a Kaiser test [45]. After the peptide synthesis, the resin was washed: DMF (7 × 1 min), DCM (3 × 1 min), and MeOH (3 × 1 min) and dried in vacuo. The last step consisted of drying a peptydylresin in a vacuum desiccator for one day at room temperature. The side chain deprotection and cleavage of the derivatized peptides from the resin was accomplished using a solution of TFA/H_2_O/TIS (95/2.5/2.5, *v*/*v*/*v*) at room temperature for 2 h. After evaporating trifluoroacetic acid, the products were lyophilized and analysed.

### 3.5. ESI-MS and LC-MS Analysis

All ESI-MS experiments were performed on the LCMS-9030 qTOF Shimadzu (Shimadzu, Kyoto, Japan) device, equipped with a standard ESI source and the Nexera X2 system. Analysis was performed in the positive ion mode between 100–3000 *m*/*z*. LCMS-9030 parameters: the nebulizing gas was nitrogen, the nebulizing gas flow was 3.0 L/min, the drying gas flow was 10 L/min, the heating gas flow was 10 L/min, interface temperature was 300 °C, desolvation line temperature was 400 °C, detector voltage was 2.02 kV, interface voltage was 4.0 kV, collision gas was argon, mobile phase (A) was H_2_O + 0.1% HCOOH, (B) was MeCN + 0.1% HCOOH, and mobile phase total flow was 0.3 mL/min. The injection volume was optimized depending on the intensity of the signals observed on the mass spectrum within the range of 0.1 to 1 μL. The singly charged ([M + H]^+^) and protonated ([M + 2H]^2+^) precursor ions were selected on the quadrupole and subsequently fragmented in the collision cell. Argon was used as a collision gas. The obtained fragments were registered as an MS/MS (tandem mass spectrometry) spectrum. The collision energy (10–30 V) was optimized for the best fragmentation. All obtained signals had a mass accuracy error in the range of 1 ppm. The LC-MS analysis was performed on an XB-C18 column (100 × 2.1 mm, 3.6 μm, Phenomenex) with the following mobile phases: (A) H_2_O + 0.1% HCOOH, (B) MeCN + 0.1% HCOOH, and the mobile phase total flow was 0.2 mL/min. Gradient conditions were from 0 to 40% B in 20 min. All the used solvents were of LC-MS grade. The obtained data were analyzed by LabSolutions software (Shimadzu, Kyoto, Japan).

### 3.6. HPLC Analysis

All products were characterized using the analytical HPLC Thermo Separation system with UV detection (210 nm) with a YMC-Pack RP C18 column (Piscataway, NJ, USA) (4.6 × 250 mm, 5 μm) and with a gradient elution of 0–40% B in A (A = 0.1% TFA in water; B = 0.1% TFA in acetonitrile/H_2_O, 4:1) over 30 min (flow rate 1 mL/min). The main fraction, corresponding to the peptide, was collected and lyophilized.

## 4. Conclusions

In the present study, we have examined the reactivity of the 5-amino-3-methyl-isoxazole-4-carboxylic acid as a non-proteinogenic amino acid in the peptide synthesis reaction on a solid support. After the synthesis of the model peptides with the following sequences, H-DVYT-NH_2_, H-EAAA-NH_2_, H-PPPP-NH_2_, and H-PPPPP-NH_2_, we modified their *N*-terminus with the 5-amino-3-methyl-isoxazole-4-carboxylic acid, obtaining a new, previously not described in the literature α/β-mixed peptide hybrid. We have characterized each of the obtained derivatives using the MS, MS/MS, and LC-MS methods. It has been shown that the amino group of the 5-amino-3-methyl-isoxazole-4-carboxylic acid remains unreactive in the formation of the Fmoc-protected derivative in typical reaction conditions. We have determined that it is possible to couple an unblocked AMIA amino acid to the α-amino group of the *N*-terminal amino acid residue present in a peptide supported on a solid support. Although the derivatization of the β-amino group of the 5-amino-3-methyl-isoxazole-4-carboxylic acid linked to a peptide was repeated many times, and we tried to modify the process conditions, it resulted in obtaining the low efficiency of the modification of this group in the coupling reaction. The obtained results indicate the possibility of including AMIA in the synthesis of new peptide derivatives on a solid support, which opens new perspectives on the use of this novel β-amino acid as a substrate in the preparation of new peptidomimetics and to exam their potential biological activity.

## Figures and Tables

**Table 1:** MS data for the model peptides.

Nr.	Peptide Sequence	[M + H]^+^ Found	[M + H]^+^ Calc.	[M + Na]^+^ Found	[M + Na]^+^ Calc.
1	H-AMIA-DVYT-NH_2_	620.267	620.267	642.250	642.249
2	H-AMIA-EAAA-NH_2_	484.215	484.215	506.197	506.197
3	H-AMIA-PPPP-NH_2_	530.276	530.272	552.258	552.254
4	H-A-AMIA-PPPPP-NH_2_	698.364	698.362	-	-

## Data Availability

Not applicable.

## Sample Availability

Samples of the described compounds are available from the authors.
